# The Temporal Order of Word Presentation Modulates the Amplitudes of P2 and N400 during Recognition of Causal Relations

**DOI:** 10.3389/fpsyg.2016.01890

**Published:** 2016-12-02

**Authors:** Xiuling Liang, Feng Xiao, Lijun Wu, Qingfei Chen, Yi Lei, Hong Li

**Affiliations:** ^1^Institute for Advanced Study, Chengdu UniversityChengdu, China; ^2^Research Centre for Brain Function and Psychological Science, Shenzhen UniversityShenzhen, China; ^3^Department of Teacher Education, Shanxi Normal UniversityLinfen, China; ^4^Department of Economics and Trade, Guangdong University of FinanceGuangzhou, China; ^5^China Center for Special Economic Zone Research, Shenzhen UniversityShenzhen, China

**Keywords:** causal relations, hierarchical relations, temporal order, P2, N400

## Abstract

The processing of causal relations has been constantly found to be asymmetrical once the roles of cause and effect are assigned to objects in interactions. We used a relationship recognition paradigm and recorded electroencephalographic (EEG) signals to explore the neural mechanism underlying the asymmetrical representations of causal relations in semantic memory. The results revealed that the verification of causal relations is faster if two words appear in “cause-effect” order (e.g., virus-epidemic) than if they appear in “effect-cause” order (e.g., epidemic-virus), whereas no such asymmetrical representation was found for the verification of hierarchical relations with reverse orders (e.g., bird-sparrow vs. sparrow-bird) in Experiment 1. Furthermore, the P2 amplitude elicited by “superordinate-subordinate” order was larger than that when in reverse order, whereas the N400 effect elicited by “cause-effect” order was smaller (more positive) than when in reverse order. However, no such asymmetry, as well as P2 and N400 components, were observed when verifying the existence of a general associative relation in Experiment 2. We suggested that the smaller N400 in cause-effect order indicates their increased salience in semantic memory relative to the effect-cause order. These results provide evidence for dissociable neural processes, which are related to role binding, contributing to the generation of causal asymmetry.

## Introduction

The ability to perceive and interpret causal relations apparent in the dynamic world is a fundamental ability of the human mind. However, there is a pervasive and fundamental bias in human understanding of causal relations. That is, once the objects representing cause and effect were presented in the environment, the strength and importance of the cause object tended to be overestimated and the effect object tended to be underestimated, and the cause and effect have certain non-interchangeable binding roles (Pearl, 2000; Fenker et al., 2005; Satpute et al., 2005; White, 2006). In fact, numerous studies have found that the causal relations are inherently asymmetrical (Fenker et al., 2005; White, 2006; Barr, 2010). Specifically, researchers have examined the causal asymmetry in several domains, such as causal perception, reasoning about Newton's third law, and causal judgment from contingency information (White, 2006). For example, two small objects, A and B, are separated by several centimeters: A moves toward B until they are in contact, at which point A stops and B starts moving along the same path (Scholl and Tremoulet, 2000). In this scenario, the impetus of A tended to be overestimated and the resistance of B tended to be underestimated, and the motion of B was often reported as caused by A, whereas few people reported the stopping of A as having been caused by B.

Most crucially, some causal asymmetries are tied to the representation of causal relationships in semantic memory. For example, lighting can cause fire, but fire cannot cause lighting. Unlike causal relations, however, if one reverses the order of the general associatively related words, such as glass and window, the terms still derive the same result. Recently, several studies have begun to explore this issue (Fenker et al., 2005; Barr, 2010; Chen et al., 2014a). For example, Fenker et al. (2005) found that participants were faster when answering about the existence of a causal relation when the causally related words were presented in the cause-effect order (e.g., *epidemic-virus*) than vice versa (e.g., *virus-epidemic*). However, no such RT (reaction time) advantage was observed when participants were asked if a general associative relationship could exist between the same word pairs. Recently, Chen et al. (2014a) found that causal relationships were verified faster if “cause” appeared vertically above “effect” than the reverse, as well as when cause horizontally preceded effect rather than the reverse. However, the hierarchical relationships were verified faster only when the superordinate concepts appeared vertically above subordinate concepts rather than the reverse. These results suggested that causal relationships were distinct from associative, and hierarchical, relationships, and the processing of causal relationships might involve additional processing, such as time priority and the distinction between cause and effect roles.

Overall, although previous studies provided compelling evidence that causal asymmetry was a pervasive and fundamental bias in human thinking, it provided few tests of the neural basis of representations underlying causal asymmetry. Moreover, the lack of a control asymmetrically associated relationship makes any inferences about the RT advantage of cause-effect order relative to effect-cause order possible (Barr, 2010). In fact, hierarchical relationships were another type of asymmetric relationship, perhaps induced by the asymmetry of the roles of “category” and “instance” (Chen et al., 2014a). That is, the nodes of superordinate concepts included the nodes of subordinate concepts, but not *vice versa* (Collins and Quillian, 1969; Rosch et al., 1976). Furthermore, the strength of statistical contingencies between items might be different (Fenker et al., 2005). For example, superordinate concepts would occur 70 times if the subordinate concepts occurred 100 times, whereas subordinate concepts would only occur 40 times if the superordinate concepts occurred 100 times, because there are more subordinate concepts. Thus, the hierarchical relationships might be used as a control condition the better to explore the nature of causal asymmetry.

As summarized by Luck (2005), event related potentials (ERPs) allowed us to determine, more directly, the stages of processing affected by stimulus manipulations. Accordingly, ERPs could be fruitful in their contribution to our understanding of causal asymmetry, incomplementary conjunction with behavior studies. As such, the N400 component might be a good physiological index for exploring this issue. Specifically, it is thought that the amplitude of the N400 component was sensitive to the strength of semantic relationships, as well as different types of semantic relationship, such as thematic vs. causal relationships (Kutas and Hillyard, 1980; Kuperberg et al., 2011; Kutas and Federmeier, 2011; Paczynski and Kuperberg, 2012; Chen et al., 2015; Wamain et al., 2015). For example, when participants were required to assess whether the relationship between subsequently presented words matched the initial causal cue, the N400 was smallest for causally related words, greater for associatively related words, and biggest for unrelated words, while keeping the level of semantic association constant across all tested conditions (Chen et al., 2015). Furthermore, studies have tried to link the N400 component to specific cognitive functions, such as prediction processing and role binding (Van Berkum et al., 2005; Kutas and Federmeier, 2011; Rabovsky and McRae, 2014). For example, when participants were required to respond to words preceding predicted targets (e.g., function words, adjectives), N400 reductions was found when the words matched, as opposed to mismatched, in gender with the predicted target (Van Berkum et al., 2005; Kutas and Federmeier, 2011).

The goal of this study was to explore the asymmetrical representations of causal relationships via ERPs in a relationship verification paradigm. To further explore this issue, we also compared the causal asymmetry with hierarchical asymmetry and found that the strength of statistical contingency from subordinate concepts to superordinate concepts is higher than the reverse. Hierarchically related words were used as the control stimuli rather than general associatively related words to prevent participants from being able to use association as a cue to causality. Specifically, in Experiment 1, participants assessed whether pairs of words were causally related in one list or hierarchically related in another list after controlling the association strengths between two orders. In Experiment 2, however, participants were required to assess whether the same pairs of words were generally associatively related. In the present study, all of the hierarchically and causally related word pairs, as well as the unrelated word pairs, were presented twice, to prevent a lack of sufficient numbers of appropriate pair types and to ensure no effect of order (Chen et al., 2014b; Liang et al., 2015). If the asymmetrical representations of hierarchical relationships were similar to causal relationships, similar pattern of results should be found. Furthermore, based on a previous study (Fenker et al., 2005), the RT advantage for cause-effect order relative to effect-cause order should be observed only for evaluation of causal relationships in Experiment 1, but not for evaluation of associative relationships in Experiment 2.

We also explored this issue with an analysis of the ERPs. If participants have noticed the strength of semantic association, the N400 component elicited by unrelated words should be consistently larger than with related words (Kutas and Federmeier, 2011). Furthermore, the verification of causal relationships should be facilitated in cause-effect order than the reverse order, and the N400 component elicited by cause-effect order should be smaller than for effect-cause order in Experiment 1, because the cause-and-effect sequences have an exclusive association relative to effect-and-cause sequences (Hume, 1748; Denkinger and Koutstaal, 2014), and the evaluations of causal relationships require a representation in which each event is mapped to specific roles of the cause or the effect. However, such an effect would not be found without specific verification of causal relationships in Experiment 2, because no such mapping process was required for the evaluations of general associative relationships (Fenker et al., 2005).

## Materials and methods

### Participants

Thirty-four healthy subjects participated in the main study, which comprised two separate experiments. Sixteen (nine males) healthy undergraduate students in Experiment 1, and eighteen healthy undergraduate students (ten females) in Experiment 2 were paid to participate in the main study. The participants that initially rated the material and recruited in Experiment 1 did not participate in Experiment 2. All participants were right handed with normal, or corrected to normal, vision between the ages of 18 and 24. They gave their informed written consent before participating in the study. The study was approved by the research ethics committee of Shenzhen University of China and was conducted in accordance with the Declaration of Helsinki. Data from one participant in Experiment 1 were discarded due to excessive EEG artifacts.

### Materials (experiments 1, 2)

Based on previous studies and the results of a series of norming studies (Fenker et al., 2005; Liang et al., 2015), 240 Chinese words (40 causally related, 40 hierarchically related, and 40 unrelated word pairs) with two-syllable words (each Chinese character corresponds to one syllable) were used in Experiments 1, 2 (See Tables S1, S2). The mean strengths and statistical frequency and standard deviations over subjects and stimuli were showed in Table 1.

**Table 1:** **The mean strengths and statistical frequency and standard deviations over subjects and stimuli**.

		**Strength**			**Statistical frequency ratings**		
		***M***	***SD***		***M***	***SD***	
			**Subjects**	**Items**		**Subjects**	**Items**
Causally	S1S2	5.46	0.76	0.50	58.15	12.32	9.60
related	S2S1	5.38	0.81	0.41	57.63	11.52	9.09
Hierarchically	S1S2	5.58	1.01	0.64	56.60	10.47	9.13
related	S2S1	5.64	0.84	0.64	62.10	14.16	9.12
Semantic	S1S2	1.51	0.25	0.25	21.27	15.72	6.24
unrelated	S2S1	1.52	0.38	0.33	20.95	11.67	6.06

### Procedure

#### Normative studies

Based on previous studies (Fenker et al., 2005), 50 hierarchically related (e.g., bird-sparrow), 50 causally related (e.g., acid–corrosion) and 50 unrelated (e.g., mile–apron) word pairs were selected and translated into Chinese. Furthermore, to increase the rate of unrelated word pairs, we created a filler condition to account for stimulus balancing, in which the related word pairs were repaired to form another sub-list of 100 unrelated pairs (50 word pairs for hierarchically related condition, e.g., fish–pine, and 50 word pairs for causally related condition, e.g., diet-tide). Subsequently, 59 healthy undergraduate students were recruited and paid to participate in several normative studies, which might affect the asymmetrical representations of causal, and hierarchical, relationships in semantic memory.

In a preliminary phase, 13 participants were required to mark any words that they had not heard before. Words that were marked by two or more subjects were removed.

After this, another 23 undergraduates participated in an associative strength test for the above 150 word pairs, the order of each word pair was counterbalanced (S1S2 vs. S2S1). In the hierarchical strength test, participants were asked to rate the degree to which the object or event described by the first word included or belonged to the object or event described by the second word on a seven-point scale, where 7 indicated the highest likelihood (Chen et al., 2014a). In the causal strength test, participants were required to rate the likelihood that the object or event described by the first word caused, or be caused by, the object or event described by the second word. The unrelated word pairs were rated on the strength of general associative relationship, in which participants were required to rate the strength of the meaningful relationship between the two words. For example, the word pair “bird-sparrow” and “acid–corrosion,” received a typical rating of “5” or “6” on the hierarchically and causally relatedness scale, respectively; while the word pair “mile–apron” received a typical rating of “1” or “2” on the associatively relatedness scale.

Furthermore, another norming task was conducted to rate the strength of statistical contingency between word pairs, which sometimes affects the associative strength between items (Fenker et al., 2005). That is, another 23 participants were presented with the aforementioned 150 word pairs; the order of each pair was counterbalanced. All pairs of words were presented, and participants were required to estimate that if the event or object described by the first of the two words occurred 100 times, how many times the event or object described by the second word would occur. For example, “if *virus* occurs 100 times, how often does *epidemic* occur?” Participants were required to rate co-occurrence on a scale from 0 to 100, in increments of 10.

#### Experiment 1

We mainly manipulated the types of semantic relations and the orders of the stimuli, which were presented in a within-subjects design. The items were divided into two lists. In one list, participants were presented with hierarchically related, unrelated and filler words, and required to decide whether the word pairs were hierarchically related or not. In the second list, participants were presented with causally related, unrelated and filler words, and required to decide whether the word pairs were causally related or not. The order of the lists and the order of the stimuli within a list were randomized and counterbalanced. The stimuli (2-syllable words) subtending approximately 2° visual angle were presented throughout the experiment.

The stimuli were presented on gray background using E-prime software. All related word pairs were repeated two times due to the lack of a sufficient number of appropriate pair types, and the orders of them were counterbalanced. To balance the stimulus, the unrelated and the filler unrelated word pairs were presented twice in each list, the order of them was also counterbalanced. In total, 640 trials were used in this study. These trials were distributed as follows: 80 causal-effect trials, 80 effect-causal trials, 80 superordinate-subordinate trials, 80 subordinate-superordinate trials, 80 unrelated S2–S1 trials, 80 unrelated S1–S2 trials, 80 filler unrelated S1–S2 trials, and 80 filler unrelated S2–S1 trials.

**Figure 1:**
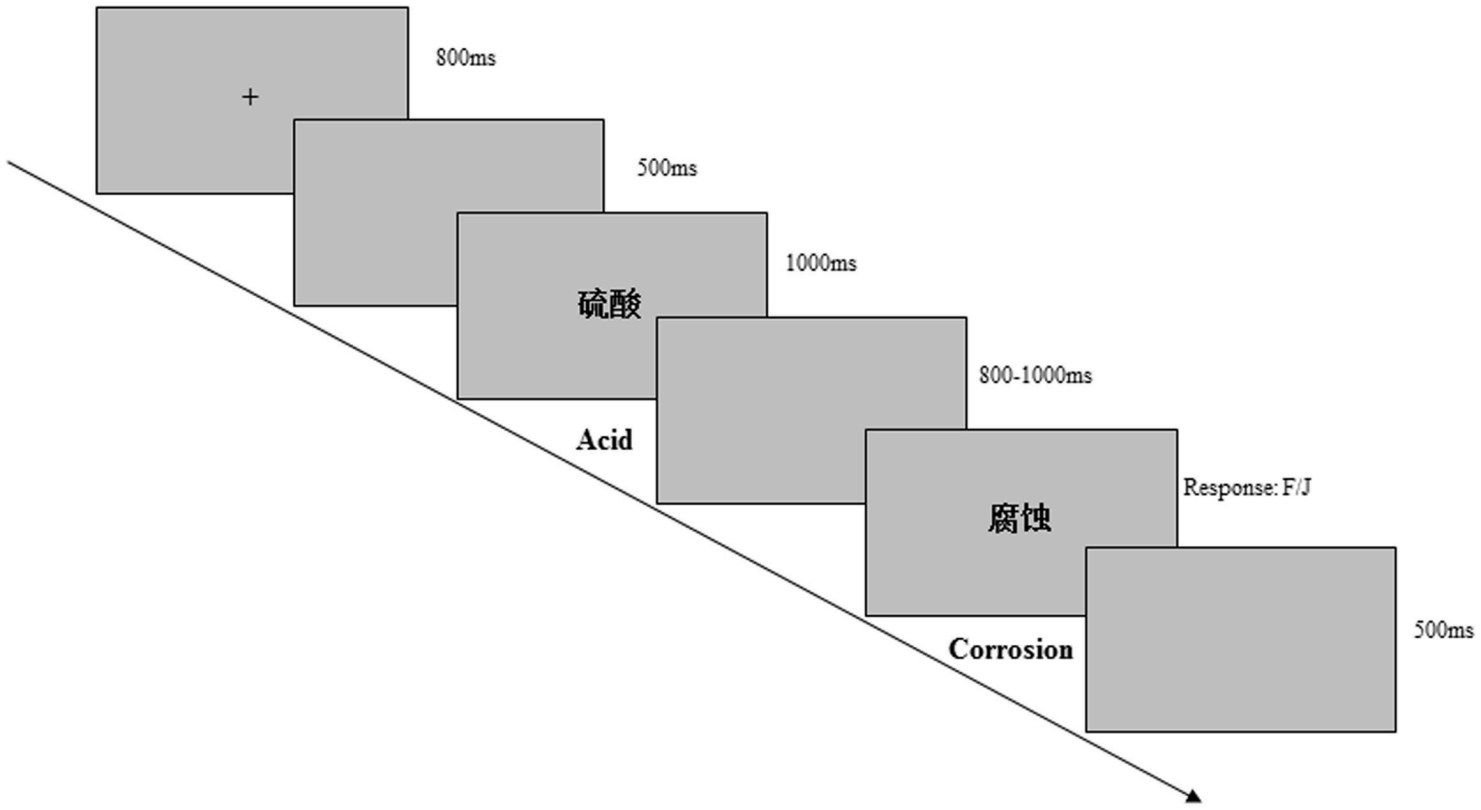
**Illustration of the experimental procedure (causally related condition)**.

The participants were shown written instructions, and all the stimuli were black. As shown in Figure 1, a fixation mark (“+”) was presented in the center of a gray screen for 800 ms at the beginning of each trial. Subsequently, S1 was presented for 1000 ms, followed by a blank screen with random duration (800–1000 ms). Next, S2 appeared on the screen and remained until participants made a response. Subjects were instructed to respond rapidly and accurately to S2, and make a “yes” or “no” response by pressing one of two keys (“F” or “J”) with the left or right index finger. The use of “F” and “J” for “yes” or “no” response was counterbalanced across subjects. Participants were informed that the existence of a causal or hierarchal relation was independent of the order of the item pairs. To make it clear that participants understood the instructions, the participants were asked to repeat the instructions in their own words. Furthermore, the participants were familiarized with the procedure through use of sixteen practice trails, which were selected from the 30 unused word pairs that were not included in the primary experiment.

#### Experiment 2

The procedure of the Experiment 2 was identical to that of Experiment 1 (Figure 1): The only difference was that participants were required to judge whether the word pairs presented within blocks was related in any way, and make an “F” or “J” response.

### ERP recordings and data analysis

Brain electrical activity was recorded from 64 tin electrodes mounted on an elastic cap based on the extended 10/20 system (Brain Products, GmbH, Germany; pass band: 0.05–100 Hz, sampling rate: 500 Hz), with a ground electrode on the medial frontal line and references on the left and right mastoid (Luck, 2005; Keil et al., 2014). The vertical electro-oculograms (EOGs) were recorded from the left eye both supra-orbitally, and infra-orbitally. The horizontal EOG was recorded from the orbital rim of both eyes. All impedances were maintained at below 10 kΩ. All the bioelectric signals were analyzed off-line using Brain Vision Analyzer 2.0. The signal was passed through a 0.1 to 35 Hz digital band-pass filter for off-line analysis. Artifacts such as blinks and eye movements were eliminated off-line using ocular correction ICA.

Averaged ERPs were also time-locked to the onset of S1 and S2. Epochs from 200 ms pre-stimulus to 1000 ms post-stimulus were extracted, segmented, baseline-corrected, and averaged (baseline data taken from −200 to 0 ms). In addition, off-line computerized artifact rejection was used to eliminate trials with mean EOG (ocular movements and eye blinks), artifacts arising from amplifier clipping, bursts of electromyographic activity, or peak-to-peak deflections exceeding ± 80 μV. As a result, less than 6% of the data were lost due to artifacts, muscle potentials, and so on. Similar to our previous studies, we have labeled the word pairs as different marks when they are presented for the first time and when they are repeated in separate blocks, and there was no significant difference between them (Chen et al., 2014b, 2015). Thus, two types of stimulus were merged, which had the advantage of avoiding problems of category specificity and physical variance that are unavoidable when using large groups of words (Renoult, 2010).

As mentioned earlier, the filler category is another sub-list of unrelated word pairs. Consequently, they are merged with the data of unrelated words. All data were analyzed using SPSS 20.0. Similar to our previous study (Chen et al., 2015; Liang et al., 2015), the N400 amplitudes elicited by unrelated words were larger than causally related (*p* < 0.001) and hierarchically related words (*p* < 0.001; Bonferroni method). However, it seems that including the unrelated words (and the unrelated filler words) in the same analysis as the word order effect should have substantially diluted any possible word order effects on the responses to the related item types. As a result, based on overall averages (see Figures 3–6), two sets of three-way repeated-measures ANOVA with the order of stimuli (S1–S2 vs. S2–S1), laterality (three levels, left, middle, and right sites) and frontality (five levels, frontal: Left–F3, middle–Fz, right–F4; frontal central: Left–FC3, middle–FCz, right–FC4; central: Left–C3, middle–Cz, right–C4; central parietal: Left–CP3, middle–CPz, right–CP4; parietal: Left–P3, middle–Pz, right–P4) as repeated factors were conducted on the mean amplitude of 150–250 ms and 300–500 ms for hierarchically related and causally related conditions, respectively. For all analyses, the degrees of freedom of the *F*-ratio were corrected for violations of the sphericity assumption according to the Greenhouse-Geisser method. Furthermore, Bonferroni corrections were used for each comparison. However, repeated-measures ANOVA indicated no significant differences were found for the ERP waves elicited by S1. Thus, only the ERPs elicited by S2 were examined (Figure 3).

**Figure 2:**
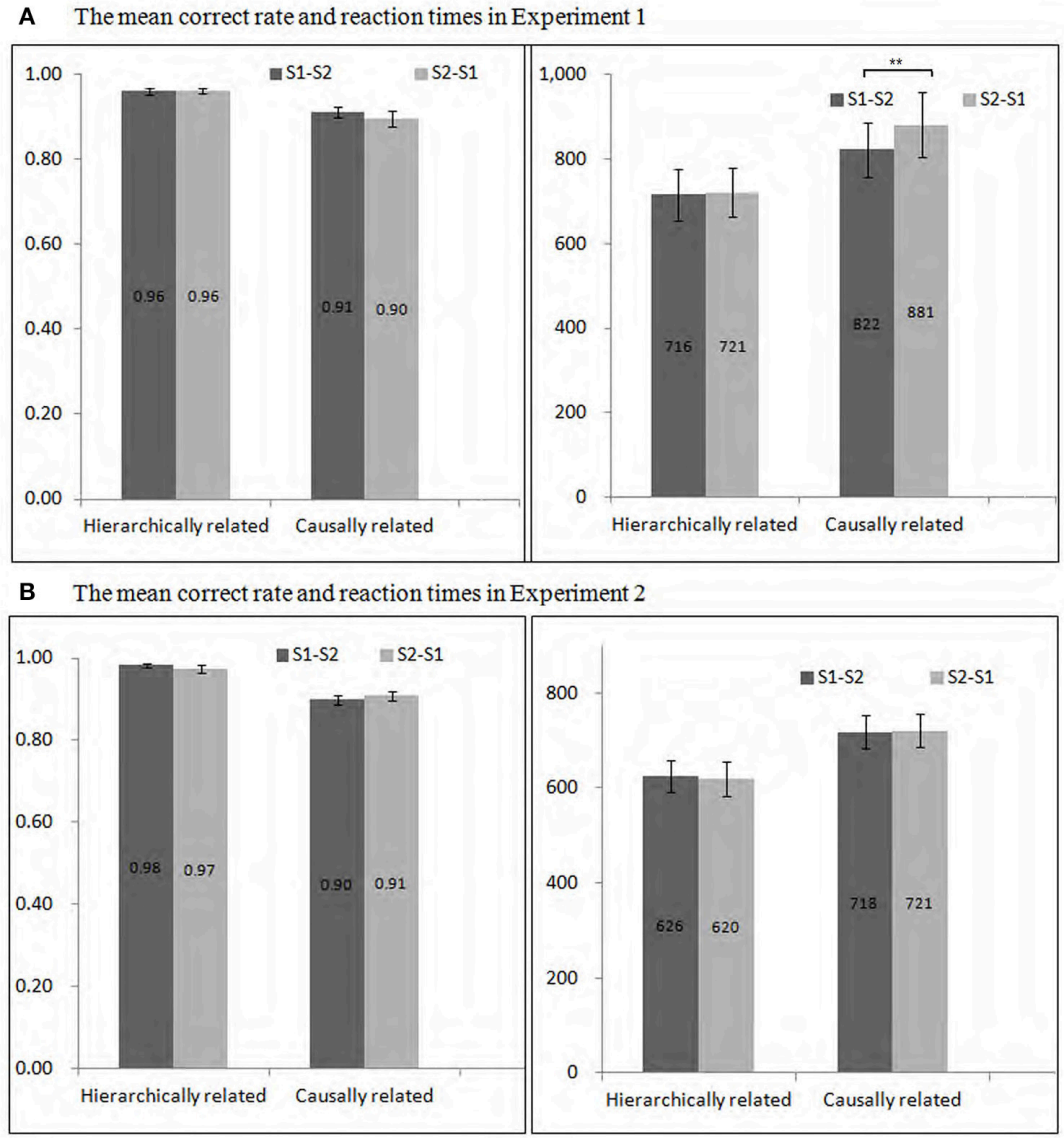
**Mean correct rate (the left, M ± SE) and reaction times (the right, M ± SE) for hierarchical, and causal, stimuli in Experiment 1 (A)** and in Experiment 2 **(B)**. The S1 in the figure represented the “superordinate level” for hierarchical related words, and “cause” for causal related words. Correspondingly, S2 represented the “subordinate level” for hierarchical related words, and “effect” for causally related words. ^**^p < 0.01.

**Figure 3:**
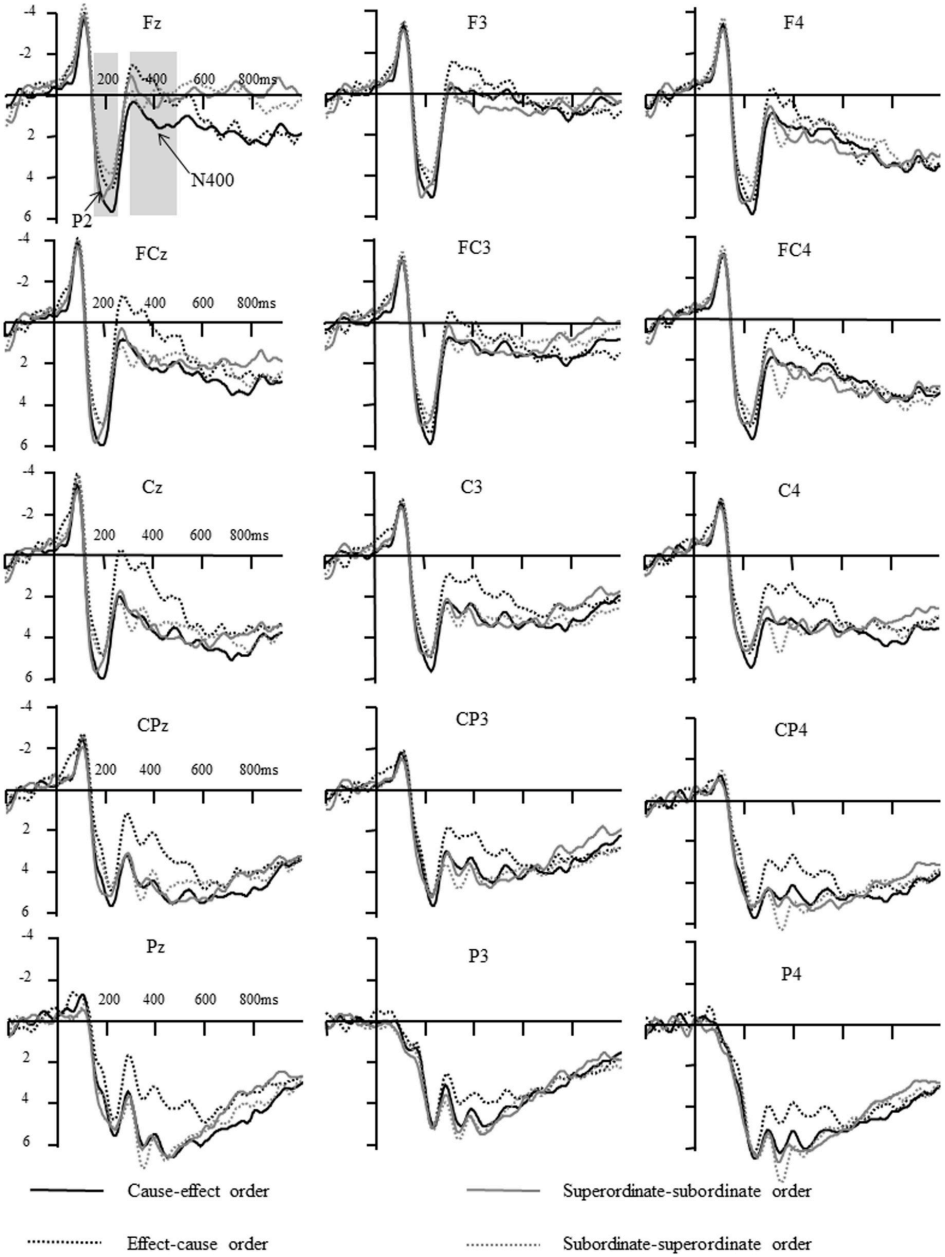
**The ERPs elicited by different conditions in causal judgments and hierarchical judgments in Experiment 1**.

**Figure 4:**
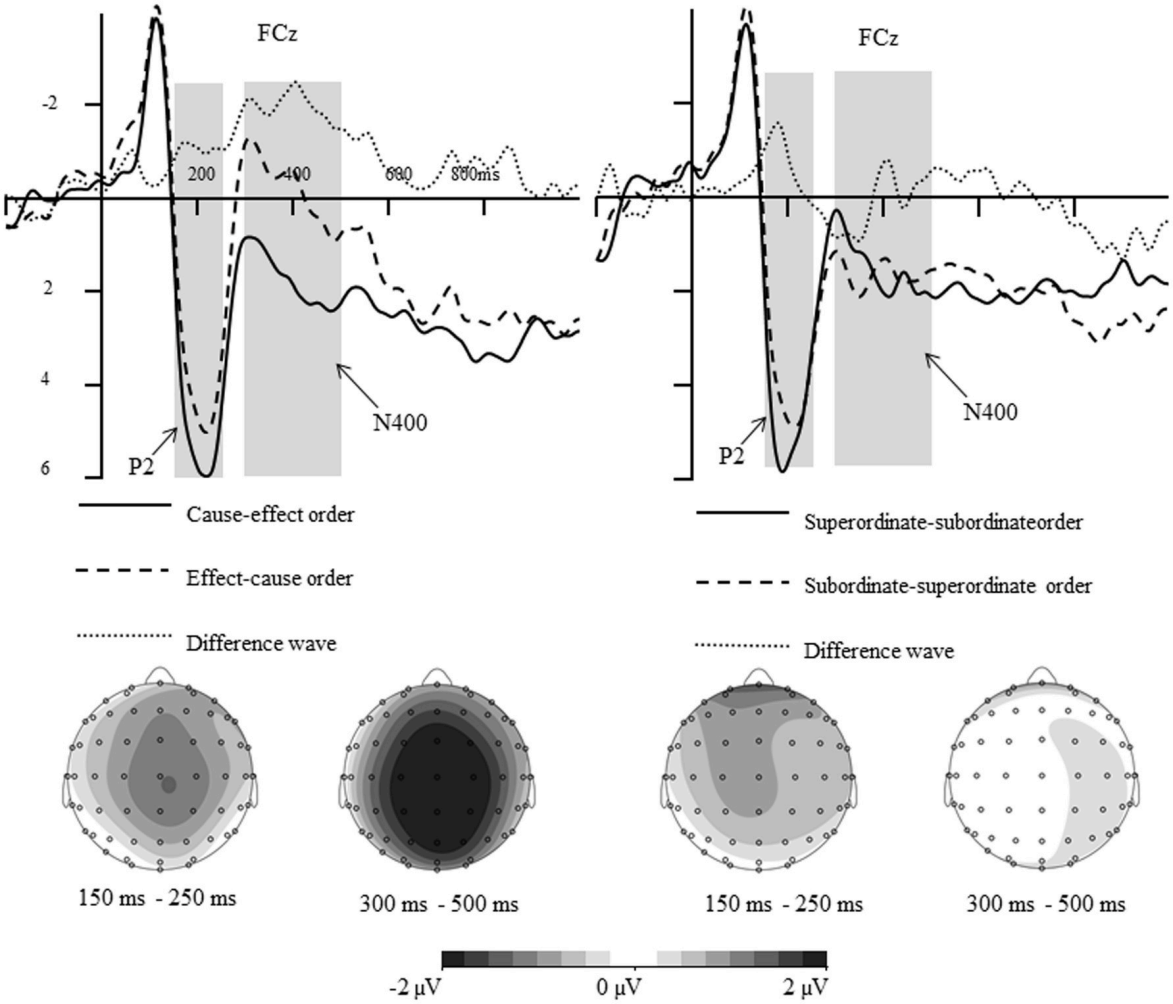
**Difference waves and topographical maps for different conditions (Left:** Effect-cause order subtracts cause-effect order for causally related stimuli; **Right:** Subordinate-superordinate level order subtracts superordinate-subordinate level order for hierarchically related stimuli).

**Figure 5:**
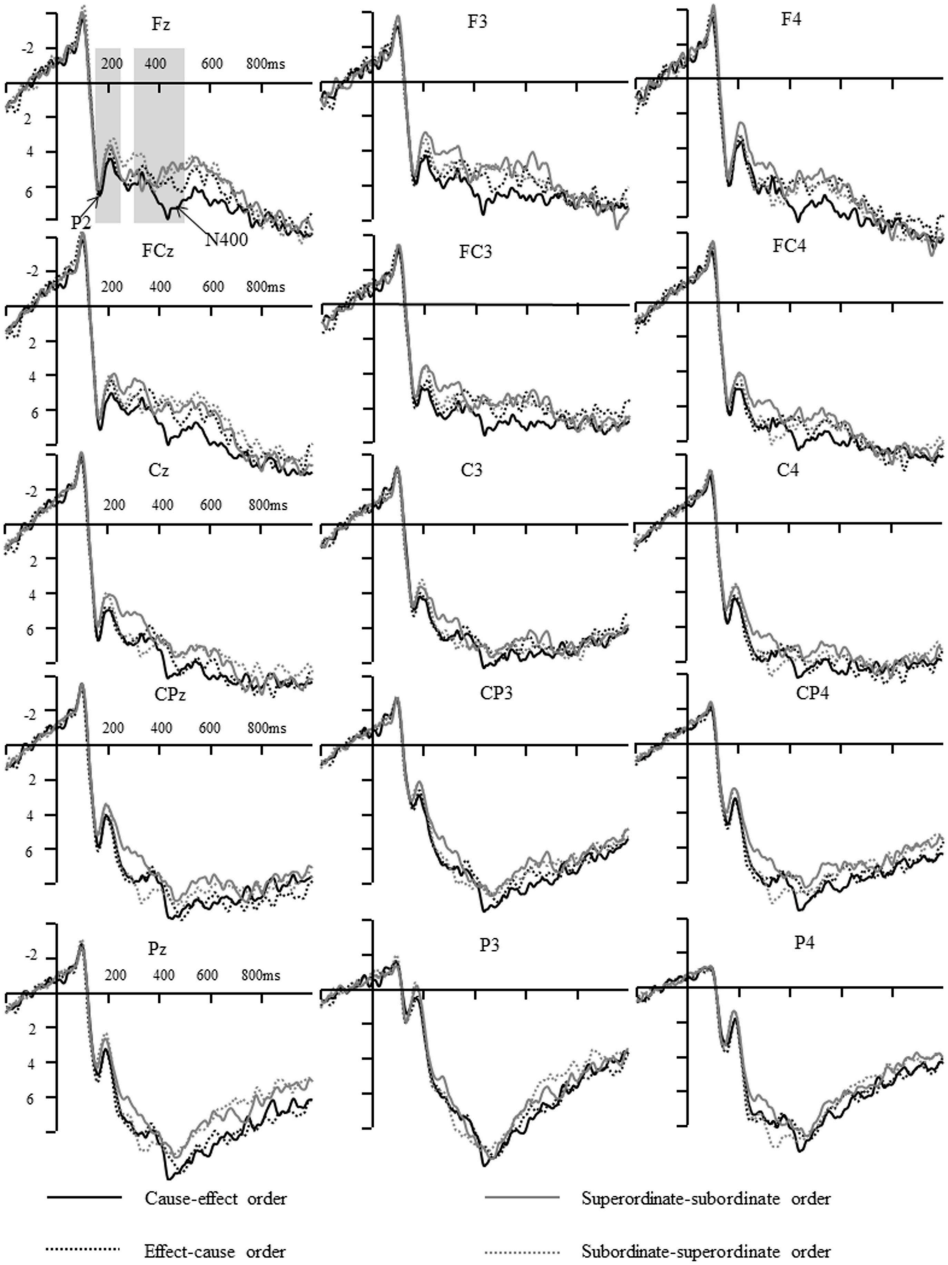
**The ERPs elicited by different conditions in general associative judgment in Experiment 2**.

**Figure 6:**
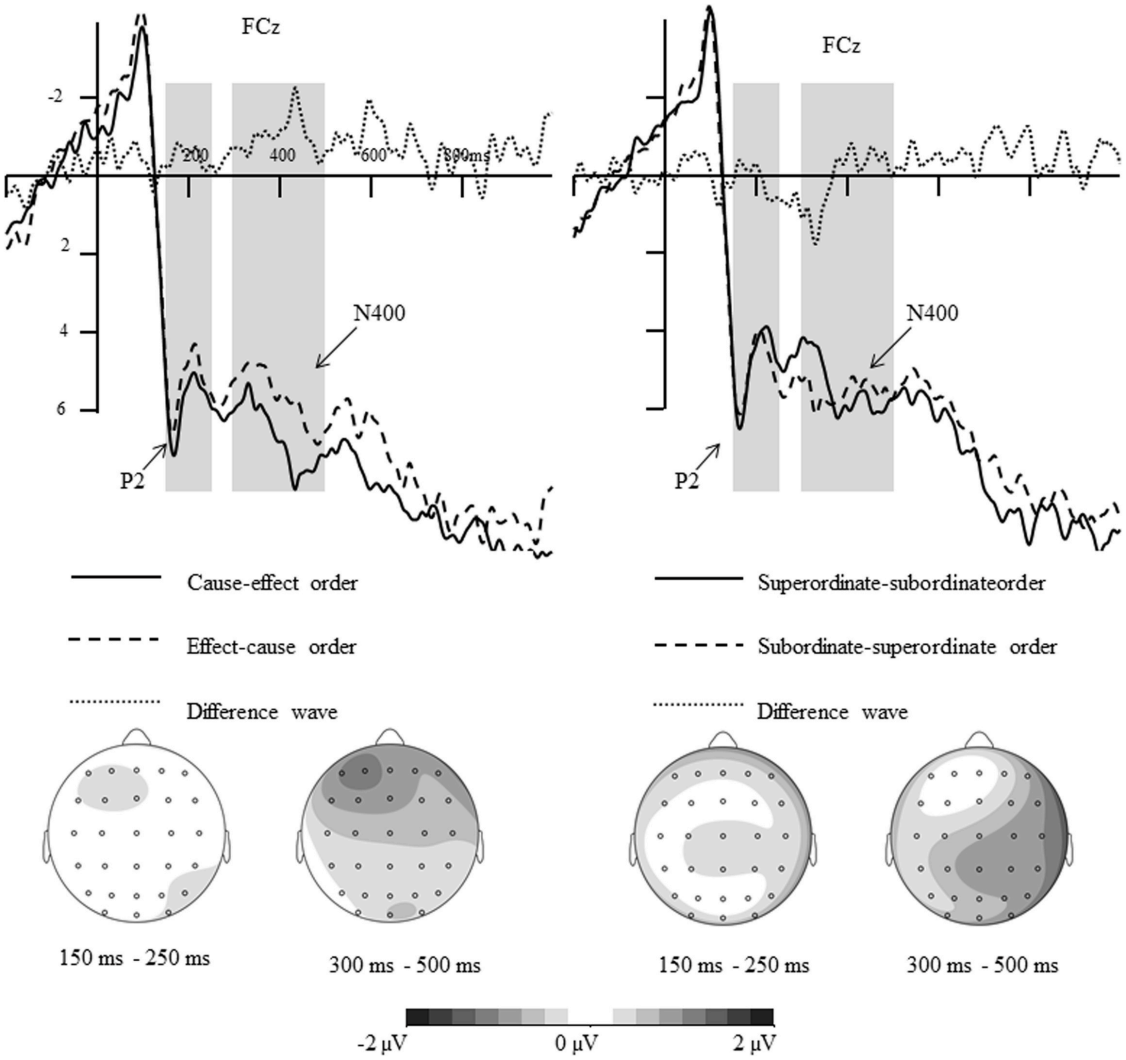
**Difference waves and topographical maps among different conditions in Experiment 2 (Left:** Effect-cause order subtracts cause-effect order; **Right:** Subordinate-superordinate level order subtracts superordinate-subordinate level order).

## Results

### The normative studies

Table 1 shows the mean strengths and standard deviations of the ratings for each condition. One way ANOVA indicate that there were significant main effects of the type of relation on the associative strength when analyzed by subjects and by items, S1S2: *F*_(2, 66)_ = 223.49, *p* < 0.001, *F*_(2, 117)_ = 899.41, *p* < 0.001; S2S1: *F*_(2, 66)_ = 242.14, *p* < 0.001, *F*_(2, 117)_ = 925.53, *p* < 0.001, respectively. The Bonferroni method was used for *post-hoc* multiple comparisons and the results indicated that the strength of unrelated word pairs was significantly lower than two related conditions (*ps* < 0.001), whereas there was no significant difference between causally related and hierarchically related word pairs, *p* > 0.10. Furthermore, the paired *t*-test suggested that there was no significant difference between S1S2 and S2S1 orders for hierarchically related, causally related and unrelated words when analyzed by subjects, *t*_(22)_ = 0.36, *p* = 0.73, *Cohen's d* = 0.06; *t*_(22)_ = 0.78, *p* = 0.44, *Cohen's d* = 0.10; and *t*_(22)_ = 0.24, *p* = 0.82, *Cohen's d* = 0.03, and by items, *t*_(39)_ = 1.38, *p* = 0.18, *Cohen's d* = 0.09; *t*_(39)_ = 1.16, *p* = 0.25, *Cohen's d* = 0.17; and *t*_(39)_ = 0.34, *p* = 0.73, *Cohen's d* = 0.03, respectively.

Similarly, one way ANOVA indicate that the main effects of type of relations on the statistical frequency ratings by subjects and by items were also significant, S1S2: *F*_(2, 66)_ = 59.04, *p* < 0.001, *F*_(2, 66)_ = 67.66, *p* < 0.001; S1S2: *F*_(2, 117)_ = 243.71, *p* < 0.001, *F*_(2, 117)_ = 302.30, *p* < 0.001. *Post-hoc* multiple comparisons indicated that the statistical frequency of unrelated word pairs was significantly lower than related conditions (*ps* < 0.001), whereas no significant difference was found between causally related and hierarchically related word pairs, *p* > 0.10. Furthermore, the paired *t*-test suggested that there was no significant difference between S1S2 and S2S1 orders in causally related and unrelated conditions when analyzed by subject, *t*_(22)_ = 0.35, *p* = 0.73, *Cohen's d* = 0.04; *t*_(22)_ = 0.25, *p* = 0.81, *Cohen's d* = 0.02; and by items, *t*_(39)_ = 0.32, *p* = 0.75, *Cohen's d* = 0.06; *t*_(39)_ = 0.28, *p* = 0.39, *Cohen's d* = 0.05. However, significant differences were found between S1S2 and S2S1 orders for hierarchically related words when analyzed by subject, *t*_(22)_ = 2.81, *p* < 0.01, *Cohen's d* = 0.44; and by items, *t*_(39)_ = 3.90, *p* < 0.001, *Cohen's d* = 0.60, respectively.

The mean word frequency, which was defined, based on a current Chinese language database (Center for Chinese Linguistics PKU, China) and key statistics pertaining to the log-transformed data was: 86 (*SD* = 0.19) for causally related words, 0.87 (*SD* = 0.16) for hierarchically related word pairs, and 0.87 (*SD* = 0.18) for unrelated words. One way ANOVA indicate that the mean frequency was not significantly different among three groups of words, *F*_(2, 237)_ = 0.05, *p* > 0.95.

### Experiment 1

#### Behavioral results

Mean correct rate (ACC) and reaction times (RTs) were presented in Figure 2A. The main effect of the order of stimuli on ACC for hierarchically related words was not significant, *F*_(1, 14)_ = 0.02, *p* = 0.905, η^2^ = 0.001. Similarly, there was no significant difference between the RTs for hierarchically related words with different orders, *F*_(1, 14)_ = 0.27, *p* = 0.611, η^2^ = 0.02.

Moreover, there was no significant difference between the ACC for the cause-effect order and effect-cause order, *F*_(1, 14)_ = 1.85, *p* = 0.195, η^2^ = 0.12. However, the main effect of the order of stimuli on RTs for causally related words was significant, *F*_(1, 14)_ = 18.29, *p* < 0.01, η^2^ = 0.57. That is, the RTs for the cause-effect order were shorter than that for the effect-cause order (*p* < 0.01).

#### ERP results

##### P2 (150–250 ms)

As show in Table 2, for the hierarchically related words, the main effect of the order of stimuli was significant, *F*_(1, 14)_ = 5.06, *p* < 0.05, η^2^ = 0.27. The Bonferroni method was used for pair-wise comparisons and the result indicated that the P2 amplitudes elicited by superordinate-subordinate order (4.39 ± 0.68 μV) were larger than subordinate-superordinate order (3.71 ± 0.71 μV).

**Table 2:** **Three-way repeated-measures ANOVA of mean amplitudes to assess the influence of temporal order on causal and hierarchical processing in Experiment 1**.

**Three-way ANOVA**	**Hierarchical processing**						**Causal processing**					
	***P2* (150–250ms)**			***N400* (300–500ms)**			***P2* (150–250ms)**			***N400* (300–500ms)**		
	***F***	***p***	***η^2^***	***F***	***p***	***η^2^***	***F***	***p***	***η^2^***	***F***	***p***	***η^2^***
Frontality	0.16	0.801	0.011	19.89^***^	0.000	0.587	0.48	0.557	0.033	16.96^***^	0.000	0.548
Laterality	0.73	0.477	0.049	5.08^*^	0.020	0.266	0.97	0.384	0.065	2.64	0.095	0.159
Order	5.06^*^	0.041	0.265	0.02	0.881	0.002	2.73	0.121	0.163	7.65^*^	0.015	0.353
Frontality ^*^ Laterality	1.17	0.334	0.077	1.05	0.374	0.070	1.62	0.178	0.104	1.04	0.394	0.069
Frontality ^*^ Order	1.61	0.225	0.103	0.48	0.546	0.033	1.58	0.227	0.102	1.16	0.319	0.077
Laterality ^*^ Order	0.77	0.432	0.052	1.49	0.245	0.096	3.13	0.080	0.182	10.03^**^	0.001	0.417
Frontality ^*^ Laterality ^*^ Order	0.35	0.830	0.024	0.63	0.619	0.043	0.41	0.771	0.028	0.53	0.698	0.037

* *p < 0.05*,

** *p < 0.01*,

*** *p < 0.001*.

For the causally related words, however, the main effect of the order of stimuli was not significant, *F*_(1, 14)_ = 2.73, *p* = 0.121, η^2^ = 0.16. That is, there was no significant difference on P2 amplitude elicited by cause-effect order (4.46 ± 0.86 μV) and effect-cause order (3.73 ± 0.65 μV).

##### N400 (300–500 ms)

For hierarchically related words, the main effect of the order of stimuli was not significant, *F*_(1, 14)_ = 0.02, *p* = 0.88, η^2^ = 0.002. The main effect of the frontality was significant, *F*_(4, 56)_ = 19.89, ε = 0.35, *p* < 0.001, η^2^ = 0.59. Pair-wise comparison indicated that the N400 amplitudes at frontal (0.69 ± 0.95 μV), frontal-central (1.74 ± 0.98 μV), and central sites (3.23 ± 0.90 μV) were larger (more negative) than that at central-parietal (4.70 ± 0.80 μV), and parietal sites (5.80 ± 0.81 μV, *ps* < 0.05). Furthermore, the N400 at frontal sites was larger than central sites (*p* < 0.01). Similarly, the main effect of laterality was significant, *F*_(3, 42)_ = 5.08, ε = 0.81, *p* < 0.05, η^2^ = 0.27. The N400 amplitude at left sites (2.71 ± 0.80 μV) was larger than that at right sites (3.87 ± 0.73 μV, *p* < 0.05). No other significant difference was found.

For the causally related words, however, the main effect of the order of stimuli was significant, *F*_(1, 14)_ = 7.65, *p* < 0.05, η^2^ = 0.35. Pair-wise comparison indicated that the N400 elicited by cause-effect order (3.13 ± 1.04 μV) was smaller than effect-cause order (1.63 ± 0.82 μV). The main effect of frontality was significant, *F*_(4, 56)_ = 16.96, ε = 0.35, *p* < 0.001, η^2^ = 0.55. Pair-wise comparison indicated that the N400 amplitudes at frontal (0.30 ± 1.05 μV) and frontal-central (1.05 ± 1.04 μV) sites were larger than that at central (2.28 ± 1.04 μV), central-parietal (3.61 ± 0.89 μV), and parietal sites (4.67 ± 0.85 μV, *ps* < 0.01). Furthermore, the N400 at central sites was larger than that at central-parietal sites (*p* = 0.029). The interaction between the order of stimuli and laterality was significant, *F*_(2, 28)_ = 10.03, ε = 0.86, *p* < 0.01, η^2^ = 0.42. Although the difference in N400 amplitude between cause-effect order and effect-cause order was found at all sites, the difference was larger at central sites (3.37 ± 1.18 μV, 1.31 ± 0.96 μV, *p* < 0.01) relative to left (2.51 ± 1.00 μV, 1.38 ± 0.75 μV, *p* < 0.05), and right (3.50 ± 1.03 μV, 2.22 ± 0.83 μV, *p* < 0.05) sites.

### Experiment 2

#### Behavioral results

Mean ACC and RTs are shown in Figure 2B. The main effect of the order of stimuli on RTs for hierarchically related words was insignificant, *F*_(1, 17)_ = 0.47, *p* = 0.501, η^2^ = 0.03. Similarly, there was no significant difference between the ACC for hierarchically related words with different orders, *F*_(1, 17)_ = 2.52, *p* = 0.131, η^2^ = 0.13.

Moreover, there was no significant difference between the RTs for the cause-effect order and effect-cause order, *F*_(1, 17)_ = 0.03, *p* = 0.856, η^2^ = 0.002. Similarly, the main effect of the type of relationship on ACC for causally related words was insignificant, *F*_(1, 17)_ = 0.96, *p* = 0.342, η^2^ = 0.05.

#### ERP results

##### P2 (150–250 ms)

As shown in Table 3, for the hierarchically related words, the main effect of the order of stimuli was insignificant, *F*_(1, 17)_ = 0.50, *p* = 0.490, η^2^ = 0.03. The main effects of the frontality and laterality were significant, *F*_(4, 68)_ = 4.22, ε = 0.35, *p* < 0.05, η^2^ = 0.20, *F*_(2, 34)_ = 4.45, ε = 0.81, *p* < 0.05, η^2^ = 0.21, respectively. The Bonferroni method was used for all pair-wise comparisons and the results indicated that the P2 amplitude at central sites (4.58 ± 0.83 μV) was larger than that at central-parietal sites (4.02 ± 0.72 μV, *p* < 0.05), and the amplitude P2 at middle sites (4.56 ± 0.85 μV) was larger than that at left sites (3.71 ± 0.69 μV, *p* < 0.05). No other significant difference was found.

**Table 3:** **Three-way repeated-measures ANOVA of mean amplitudes to assess the influence of temporal order on causal and hierarchical processing in Experiment 2**.

**Three-way ANOVA**	**Hierarchical processing**						**Causal processing**					
	***P2* (150–250 ms)**			***N400* (300–500 ms)**			***P2* (150–250 ms)**			***N400* (300–500 ms)**		
	***F***	***p***	***η^2^***	***F***	***p***	***η^2^***	***F***	***P***	***η^2^***	***F***	***p***	***η^2^***
Frontality	4.22^*^	0.036	0.199	10.83^**^	0.001	0.389	5.68^*^	0.015	0.250	10.73^**^	0.001	0.387
Laterality	4.45^*^	0.024	0.207	0.89	0.380	0.050	5.08^*^	0.018	0.230	0.72	0.450	0.041
Order	0.50	0.490	0.028	0.92	0.352	0.051	0.001	0.970	0.000	1.87	0.190	0.099
Frontality ^*^ Laterality	2.94^*^	0.040	0.147	3.12^*^	0.016	0.155	4.63^**^	0.006	0.214	2.71^*^	0.046	0.137
Frontality ^*^ Order	0.35	0.682	0.020	0.87	0.440	0.049	2.51	0.098	0.129	2.23	0.132	0.116
Laterality ^*^ Order	0.47	0.612	0.027	4.16^*^	0.030	0.197	1.05	0.347	0.058	0.06	0.883	0.003
Frontality ^*^ Laterality ^*^ Order	0.74	0.569	0.042	0.96	0.439	0.053	0.89	0.476	0.050	0.39	0.827	0.022

* *p < 0.05*,

** *p < 0.01*.

For the causally related words, similarly, the main effect of the order of stimuli was not significant, *F*_(1, 17)_ = 0.001, *p* = 0.97, η^2^ < 0.001. The main effects of the frontality and laterality were significant, *F*_(4, 68)_ = 5.68, ε = 0.37, *p* < 0.05, η^2^ = 0.25, *F*_(2, 34)_ = 5.08, ε = 0.82, *p* < 0.05, η^2^ = 0.23. Pair-wise comparison indicated that the P2 amplitude at frontal central sites (5.49 ± 0.88 μV) was larger than that at frontal sites (4.94 ± 0.93 μV, *p* < 0.05), and the P2 amplitude at central sites (5.29 ± 0.84 μV) was larger than that at parietal sites (3.84 ± 0.53 μV, *p* < 0.05). Furthermore, the P2 amplitude at middle sites (5.29 ± 0.84 μV) was larger than that at left sites (4.35 ± 0.67 μV, *p* < 0.05).

##### N400 (300–500 ms)

For the hierarchically related words, the main effect of the order of stimuli was not significant, *F*_(1, 17)_ = 0.92, *p* = 0.35, η^2^ = 0.05. Although the interaction between the order of stimuli and the laterality was significant, *F*_(2, 34)_ = 4.16, ε = 0.88, *p* < 0.05, η^2^ = 0.20, pair-wise comparison indicated there was no significant difference between different orders at left (*p* = 0.618), middle (*p* = 0.409), and right sites (*p* = 0.153). The main effect of frontality was significant, *F*_(4, 68)_ = 10.93, ε = 0.37, *p* < 0.001, η^2^ = 0.39. Pair-wise comparison indicated that the N400 amplitudes at frontal-central (6.01 ± 0.96 μV) sites were larger than that at central sites (7.14 ± 0.86 μV), central-parietal (8.04 ± 0.77 μV), and parietal sites (8.45 ± 0.53 μV, *ps* < 0.05). Furthermore, the N400 at central parietal sites was smaller than that at frontal and central sites (*ps* < 0.01). No other significant difference was found.

For the causally related words, similarly, the main effect of the order of stimuli was not significant, *F*_(1, 17)_ = 1.87, *p* = 0.19, η^2^ = 0.10. The main effect of frontality was significant, *F*_(4, 68)_ = 10.73, ε = 0.40, *p* < 0.01, η^2^ = 0.39. Pair-wise comparison indicated that the N400 amplitudes at frontal (6.12 ± 0.92 μV), and frontal-central sites (6.48 ± 0.85 μV), were larger than that at central-parietal (8.19 ± 0.78 μV) and parietal sites (8.46 ± 0.59 μV, *ps* < 0.05).

## Discussion

The main purpose of this study was to investigate the electrophysiological characteristics of causal asymmetry by recording ERPs in a relationship recognition paradigm. Significant RT advantage was found for same causally related words with different orders of presentation in Experiment 1. That is, the causal relations were accessed faster if two words appear in cause-effect order relative to in effect-cause order when assessing a causal relationship. These results were consistent with previous studies (Fenker et al., 2005; Barr, 2010), suggesting that participants have distinguished the roles of cause and effect when evaluating the presence of a causal relationship. However, such an RT advantage was not found for hierarchical relationships even at long SOA. These results were aligned with those of our previous study (Chen et al., 2014b), which found that the asymmetry representation of “category” and “member” in hierarchical relationships might be induced by other factors (e.g., the spatial arrangement), rather than temporal order.

The main finding of Experiment 2 was that when participants were asked to judge whether the identical items in Experiment 1 were associated, no RT advantage was found for cause-effect order relative to effect-cause order. Similar to previous study, these results suggested that the processing of causal judgment in Experiment 1 was dissociative from the associative judgment in Experiment 2, which might recruited additional cognitive resources, such as role binding or prediction (Fenker et al., 2005, 2010). Unlike in Experiment 1, however, our subjects did not appear to distinguish the role of cause and effect when queried about the presence of an associative relationship for the same causally related items. In other words, causal judgment might require a representation in which each word pair was mapped to specific roles of the cause or the effect, whereas no such mapping process is required for an associative judgment (Fenker et al., 2005; Chen et al., 2015).

Similar to previous studies, these results were in accordance with the causal model view, which postulated that learners can represent asymmetric causal relations explicitly and use this knowledge when learning about knowledge stored in semantic memory (Waldmann et al., 1995; Waldmann, 2000; Fenker et al., 2005; Barr, 2010; Holyoak and Cheng, 2011). Indeed, there has been considerable debate about whether this asymmetry is mirrored in human cognitive representations. The associative view interpreted the asymmetries by assuming that associations in the S1–S2 order may tend to be stronger than associations in the S2–S1 order, or *vice versa* (Shanks and Lopez, 1996; Cobos et al., 2002). According to causal model view, however, the asymmetric representation of semantic causal knowledge is, in part, determined by access to specifically causal relational knowledge (Fenker et al., 2005). Specifically, the evaluations of causal relationships require a representation in which each event is mapped to specific roles of the cause or the effect, whereas no such mapping process is required for evaluation of general associative relationships. In the present study, although the association strength was equated for the cause-effect and effect-cause orders, significant RT advantage was still found between them in Experiment 1, but not in Experiment 2. As a result, the causal model view is more reasonable in the interpretation of these results.

This interpretation is further supported by the ERP data. As shown in Figures 3, 4, when participants were required to make an explicit causal or hierarchical judgment in Experiment 1, the amplitude of P2 was sensitive to the order of hierarchically related words, whereas the amplitude of N400 was sensitive to the orders of causally related words. However, no significant differences for P2 and N400 were found between different orders when evaluating an associative relationship in Experiment 2. The divergence of P2/N400 response yielded a new insight into the asymmetric representations of causal relation and hierarchical relationships, and provided a better explanation for the differences between causal, and hierarchical, relationships.

### P2-wave amplitude predicts perceived temporal order

It is known that the P2 is involved in language processes including language context information and expectancy for a given word (Federmeier et al., 2005; Wlotko and Federmeier, 2007). For example, Federmeier and Kutas have found that the P2 effect was sensitive to the level of expectancy for a particular item in a sentence (As in: “At the zoo, my sister asked if they painted the black and white stripes on the animal. I explained to her that they were natural features of a zebra/donkey/poodle.”), which indicated that the contextual information was used in the visual analysis of upcoming stimuli. Furthermore, researchers have found that the larger P2 amplitude was elicited by words in strongly constraining contexts (e.g., The child was born with a rare disease/gift.) relative to those in weakly constraining contexts (e.g., Mary went into her room to look at her clothes/gift.), independent of whether the actual word was that expected, or not (Federmeier et al., 2005; Wlotko and Federmeier, 2007).

For the hierarchical related words, there was a main effect of the order of the stimuli on P2 amplitudes in Experiment 1. As participants were required to make an explicit hierarchical judgment in a separate block, they paid more attention to processing the specific features of hierarchical relationships. As a result, the prediction difference might involve in the processing of hierarchical relationships with different orders because the statistical frequency was different. Unlike in Experiment 1, no significant P2 effect was found for the same word pairs with different orders in Experiment 2. Maybe, when participants were required to make a general associative judgment about identical items within blocks, they mainly focused on the differences between related and unrelated relationships, rather than the features of certain relationship.

For causally related words, however, no significant difference in P2 amplitude was found between different orders in both Experiments 1, 2. These results were consistent with the prediction interpretation of hierarchically related words, because there was no significant difference in the strength of statistical contingencies between cause-effect order and effect-cause order. In other words, the processing of causal asymmetry might be different from hierarchical asymmetry, because it was in part determined by the proximity, exclusivity, and priority (Denkinger and Koutstaal, 2014). This interpretation agreed with what the linguistic P2 effect represented in the cognitive processing of prediction (Federmeier et al., 2005; Wlotko and Federmeier, 2007).

There might be a limitation when we try to explain the P2 effect from a predictive view: That is, the word pairs in the hierarchical list sometimes share a character at different positions (e.g., 
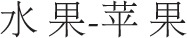
), while the causal and unrelated stimuli do not share characters. This shared feature might affect the P2 amplitude at different orders. For example, recent studies have found that orthography exerts a significant influence on the ERP effect at 150–250 ms (Hsu et al., 2009; Jouravlev et al., 2014). However, this view cannot explain why the difference in P2 effect for hierarchically related word pairs with different orders was only found in Experiment 1, but not in Experiment 2. Future research into this issue was deemed necessary.

### The N400-wave is specifically tuned to role binding

The main finding of this study was that smaller N400 component between 300 and 500 ms was elicited by cause-effect order relative to effect-cause order when assessing a causal relationship in Experiment 1. It is plausible that the N400 effect observed between cause-effect and effect-cause orders is related to the processing of causal relationships. Specifically, the smaller N400 to cause-effect order relative to the effect-cause order may indicate that judging causality requires a process called dynamic role binding (Hummel and Holyoak, 2003; Satpute et al., 2005). That is, the additional working memory might be required to form a representation of causal relations in which specific events are bound to the roles of “cause” and “effect” (Hummel and Holyoak, 2003; Satpute et al., 2005). For example, for the word pairs “virus/epidemic,” a participant needs to evaluate the specific cause and effect roles of both items when assess a causal relationship. Furthermore, as the representation of causal relationships is asymmetric (Fenker et al., 2005; Chen et al., 2014a), the verification of causal relations not only depends on sampling semantic priming-like hierarchical relationships, but also the role binding of the “cause” and “effect” events.

Another plausible interpretation of our N400 effect is that it indexes something about the prediction process (Fenker et al., 2010; Rabovsky and McRae, 2014). When participants were presented with the first word, there was a prediction process, and the prediction from “cause” to “effect” was stronger than that in the reverse order (Fenker et al., 2010; Rabovsky and McRae, 2014). In other words, when one word referring to cause was presented first, participants had an automatic tendency to infer the other word referring to effect; however, there were no such clear predictions for words in effect-cause order. Thus, the verification of causal relationships is facilitated, and elicited smaller N400, if the two words appear in “cause-effect” order than if they appear in “effect-cause” order.

We are more inclined to interpret the obtained effects to be the result of role binding, rather than the prediction process, for the following reasons: First, there was no significant difference in the strength of statistical contingency for causally related words with different orders; second, although there was a significant difference in the strength of statistical contingency for hierarchically related words with different orders, no significant N400 effects were found between them; and third, when participants were required to evaluate an associative relationship between the same causally related words in Experiment 2 which need not distinguish the roles of cause and effect (Fenker et al., 2005), no significant N400 effect was found with different orders.

As mentioned above, no significant difference in the N400 component was found for hierarchically related words with different temporal orders. In fact, these results did not contradict the above view from the perspective of N400, as no significant RT advantage was found for hierarchically related words presented in different orders. Similarly, when participants were required to make a general associative judgment about the same causally related words in Experiment 2, no significant P2 and N400 effects were found. Based on the behavior data, these results suggested that participants have distinguished the specific roles of cause and effect when verifying a causal relationship (Fenker et al., 2005; Holyoak and Cheng, 2011).

## Summary and conclusions

The present findings yielded new insights into the asymmetrical representations of causal relationships in semantic memory. According to these results, the P2 amplitude was sensitive to the order of hierarchically related words, which might reflect the processing of prediction. Furthermore, the N400 component elicited by “cause-effect” order was smaller than that for “effect-cause” order when assessing a causal relationship, which indicated that participants appear to distinguish the specific roles of cause and effect. Overall, these results suggested that role binding might be involved in the verification of causal relationships, and that the causal-model view is more suited to interpretation of the RT advantage of a causal relationship.

## Author contributions

Conceived and designed the experiments: XL, QC, and HL; Performed the experiments: XL and LW; Analyzed the data: XL, QC, and FX; Wrote the paper: XL, YL, and QC.

### Conflict of interest statement

The authors declare that the research was conducted in the absence of any commercial or financial relationships that could be construed as a potential conflict of interest.
