# Supplementary material for: The Temporal Order of Word Presentation Modulates the Amplitudes of P2 and N400 during Recognition of Causal Relations
Source: Front Psychol. 2016 Dec 2;7:1890. doi: 10.3389/fpsyg.2016.01890 (PMC5134357; doi:10.3389/fpsyg.2016.01890)
Supplement: Supplementary file 1 [file Table1.docx]

**S1 Table.** Normed Causally Related and Hierarchically Related Word Pairs Used in the Experiments.

| **Causally related word pairs** | | **English translations** | | **Hierarchically related word pairs** | | **English translations** | |
| --- | --- | --- | --- | --- | --- | --- | --- |
| 硫酸 | 腐蚀 | acid | corrosion | 衣物 | 夹克 | clothes | jacket |
| 酒精 | 事故 | alcohol | accident | 工具 | 铁锤 | tools | hammer |
| 攻击 | 防御 | attack | defense | 花卉 | 牡丹 | flower | peony |
| 细菌 | 感染 | bacteria | infection | 珠宝 | 项链 | jewelry | necklace |
| 巨响 | 耳聋 | bang | deafness | 食物 | 土豆 | food | potatoes |
| 敲打 | 伤痕 | beat | bruise | 住宅 | 公寓 | tenement | flats |
| 背叛 | 怀疑 | betrayal | distrust | 疾病 | 麻疹 | disease | measles |
| 犯罪 | 逮捕 | crime | arrest | 躯体 | 手臂 | body | arm |
| 疾病 | 注射 | disease | injection | 调料 | 酱油 | condiment | sauce |
| 节食 | 饥饿 | diet | hunger | 行星 | 火星 | planet | mars |
| 干旱 | 饥荒 | drought | famine | 宠物 | 小狗 | pet | puppy |
| 药品 | 止痛 | drug | relief | 鞋袜 | 球鞋 | footwear | sneakers |
| 间谍 | 叛国 | espionage | treason | 酒类 | 啤酒 | liquor | beer |
| 肥料 | 生长 | fertilizer | growth | 鱼类 | 金鱼 | fish | goldfish |
| 黑帮 | 暴乱 | gang | riot | 蘑菇 | 香菇 | fungus | mushrooms |
| 燃气 | 爆炸 | gases | explosion | 鸟类 | 鹦鹉 | birds | parrot |
| 基因 | 秃顶 | genes | baldness | 仪器 | 天平 | instrument | scales |
| 黄金 | 财富 | gold | wealth | 体操 | 双杠 | gymnastics | parallel Bars |
| 月球 | 潮汐 | moon | tide | 药材 | 人参 | medicinal material | ginseng |
| 损伤 | 疤痕 | lesion | scar | 昆虫 | 甲虫 | insect | beetle |
| 闪电 | 火灾 | lightning | fire | 油脂 | 菜油 | grease | rape oil |
| 高温 | 汗水 | humidity | sweat | 电器 | 冰箱 | domestic appliance | refrigerator |
| 疾病 | 治疗 | illness | treatment | 容器 | 水桶 | container | bucket |
| 磁铁 | 吸引 | magnet | attraction | 水果 | 苹果 | fruit | apple |
| 笑话 | 逗乐 | joke | amusement | 家具 | 沙发 | furniture | sofa |
| 鬼片 | 噩梦 | movie | nightmare | 厨具 | 汤勺 | kitchen ware | spoon |
| 变异 | 癌症 | mutation | cancer | 蔬菜 | 白菜 | vegetable | cabbage |
| 定购 | 发货 | order | delivery | 车辆 | 货车 | vehicle | truck |
| 恐慌 | 逃走 | panic | escape | 武器 | 手枪 | weapon | gun |
| 悲伤 | 哭泣 | sadness | crying | 乐器 | 钢琴 | musical instrument | piano |
| 食盐 | 口渴 | salt | thirst | 文具 | 钢笔 | stationery | pen |
| 刮伤 | 献血 | scratch | blood | 媒体 | 广播 | media | broadcast |
| 震惊 | 尖叫 | shock | scream | 树木 | 樟树 | trees | camphor tree |
| 扭伤 | 红肿 | sprain | swell | 饮料 | 橙汁 | drink | orange juice |
| 压力 | 疲劳 | stress | fatigue | 器官 | 心脏 | organ | heart |
| 糖果 | 龋齿 | sweets | cavity | 茶叶 | 绿茶 | tea | green tea |
| 锻炼 | 健康 | training | fitness | 影视 | 电影 | film and TV | film |
| 垃圾 | 臭味 | trash | stink | 家禽 | 母鸡 | poultry | hen |
| 重伤 | 昏迷 | trauma | coma | 田径 | 长跑 | track and field | long-distance race |
| 病毒 | 瘟疫 | virus | epidemic | 球类 | 足球 | footwear | football |

**S2 Table.** Normed Unrelated Word Pairs Used in the Experiments.

| **Unrelated words** | | **English translations** | | **Unrelated words** | | **English translations** | |
| --- | --- | --- | --- | --- | --- | --- | --- |
| 形状 | 铝材 | shape | aluminum | 盆地 | 学院 | basin | academy |
| 制服 | 插头 | smock | plug | 篮子 | 风筝 | basket | kite |
| 树木 | 女仆 | tree | maid | 画笔 | 骰子 | brush | dices |
| 钻石 | 齿轮 | diamond | gear | 气泡 | 丝绒 | bubble | velvet |
| 唱片 | 地面 | disk | ground | 厨师 | 害怕 | chef | fear |
| 钻孔 | 客人 | drill | guest | 小丑 | 地图 | clown | map |
| 老鹰 | 儿童 | eagle | child | 玻璃 | 急流 | glass | rush |
| 鸡蛋 | 说谎 | eggs | liar | 草地 | 拳头 | grass | fist |
| 引擎 | 手套 | engine | glove | 体操 | 蔬菜 | gymnastic | vegetables |
| 风景 | 数学 | landscape | maths | 海港 | 车库 | harbor | garage |
| 榜样 | 曲线 | lead | curve | 保险 | 冰糕 | insurance | ice cream |
| 柠檬 | 足球 | lemon | soccer | 标点 | 女王 | point | queen |
| 豹子 | 河流 | leopard | river | 海报 | 汉堡 | posters | hamburger |
| 公里 | 围裙 | mile | apron | 背叛 | 蟑螂 | revolting | roach |
| 奇迹 | 生姜 | miracle | ginger | 饼干 | 耳朵 | cookie | nose |
| 老鼠 | 光线 | mouse | light | 沙发 | 面团 | couch | dough |
| 页码 | 礼服 | page | tuxedo | 跳舞 | 液体 | dancer | liquid |
| 父母 | 天气 | parents | weather | 小鹿 | 铅笔 | deer | pencil |
| 屋顶 | 海员 | roof | seaman | 茶水 | 图表 | tea | graph |
| 野蛮 | 机场 | savage | airport | 雨水 | 拳师 | water | boxer |
